# NDR1 increases NOTCH1 signaling activity by impairing Fbw7 mediated NICD degradation to enhance breast cancer stem cell properties

**DOI:** 10.1186/s10020-022-00480-x

**Published:** 2022-05-04

**Authors:** Ling-Ling Wang, Xiao-Yun Wan, Chun-Qi Liu, Fei-Meng Zheng

**Affiliations:** 1 Department of Medical Oncology of The Eastern Hospital, The First Affiliated Hospital, Sun Yat-Sen University, No.58, Zhong Shan Er Lu, Guangzhou, 510080 China; 2 Guangdong Provincial Key Laboratory of Orthopedics and Traumatology, The First Affiliated Hospital, Sun Yat-Sen University, Guangzhou, China; 3 Department of Medical Oncology, Guangzhou Panyu Hospital of Chinese Medicine, Guangzhou University of Chinese Medicine, Guangzhou, China; 4 Department of Thoracic Surgery, Panyu Central Hospital, Guangzhou, China

**Keywords:** NDR1, NOTCH signaling, Cancer stem cell, Fbw7, Breast cancer

## Abstract

**Background:**

The existence of breast cancer stem cells (BCSCs) causes tumor relapses, metastasis and resistance to conventional therapy in breast cancer. NDR1 kinase, a component of the Hippo pathway, plays important roles in multiple biological processes. However, its role in cancer stem cells has not been explored. The purpose of this study was to investigate the roles of NDR1 in modulating BCSCs.

**Methods:**

The apoptosis was detected by Annexin V/Propidium Iodide staining and analyzed by flow cytometry. BCSCs were detected by CD24/44 or ALDEFLUOR staining and analyzed by flow cytometry. The proliferation ability of BCSCs was evaluated by sphere formation assay. The expression of interested proteins was detected by western blot analysis. The expression of HES-1 and c-MYC was detected by real-time PCR. Notch1 signaling activation was detected by luciferase reporter assay. Protein interaction was evaluated by immunoprecipitation. Protein degradation was evaluated by ubiquitination analysis. The clinical relevance of NDR1 was analyzed by Kaplan–Meier Plotter.

**Results:**

NDR1 regulates apoptosis and drug resistance in breast cancer cells. The upregulation of NDR1 increases CD24^low^/CD44^high^ or ALDEFLUOR^high^ population and sphere-forming ability in SUM149 and MCF-7 cells, while downregulation of NDR1 induces opposite effects. NDR1 increased the expression of the Notch1 intracellular domain (NICD) and activated the transcription of its downstream target (HES-1 and c-MYC). Critically, both suppression of Notch pathway activation by DAPT treatment or downregulation of Notch1 expression by shRNA reverses NDR1 enhanced BCSC properties. Mechanically, NDR1 interactes with both NICD or Fbw7 in a kinase activity-independent manner. NDR1 reduces the proteolytic turnover of NICD by competing with Fbw7 for NICD binding, thereby leading to Notch pathway activation. Furthermore, NDR1 might function as a hub to modulate IL-6, TNF-α or Wnt3a induced activation of Notch1 signaling pathway and enrichment of breast cancer stem cells. Moreover, we find that the elevation of NDR1 expression predictes poor survival (OS, RFS, DMFS and PPS) in breast cancer.

**Conclusion:**

Our study revealed a novel function of NDR1 in regulating BCSC properties by activating the Notch pathway. These data might provide a potential strategy for eradicating BCSC to overcome tumor relapses, metastasis and drug resistance.

**Supplementary Information:**

The online version contains supplementary material available at 10.1186/s10020-022-00480-x.

## Background

Breast cancer is one of the leading causes of cancer-related deaths in women worldwide (Siegel et al. 2016). Despite the improvement in breast cancer screening and the advances in treatment strategy, the survival of breast cancer patients was severely impaired by cancer relapse and metastasis. Accumulating evidence indicates that the existence of breast cancer stem cells (BCSCs), which possess the capacity for self-renewal and differentiation, causes the tumor relapses, metastasis and resistance to conventional therapy (Ayob and Ramasamy 2018), implying that eradicating BCSCs could be a therapeutic strategy for overcoming relapses, metastasis and resistance. Thus, understanding the mechanism of BCSCs regulation will be conducive to target BCSCs.

The Notch pathway is a highly conserved developmental pathway responsible for cell fate decisions (Ehebauer et al. 2006). Exercising strict control over the activation of the Notch pathway is critical during normal tissue development (Callahan and Egan 2004). The deregulation of Notch pathway activation is implicated as a key driver in a variety of cancers (Radtke and Raj 2003). Notch pathway is activated by binding of Notch ligand, including Delta-like 1, 2, 4 and Jagged 1, 2 (D'Souza et al. 2010). The Notch ligand-receptor complex then undergoes several key proteolytic cleavages (Kopan and Ilagan 2009), including the cleavage mediated by the ADAM/TACE family of proteases at an extracellular site (S2) and the cleavage mediated by the γ-secretase complex. γ-Secretase cleavage releases the Notch intracellular domain (NICD) to translocate into the nucleus and activate CSL [CBF-1 (C-promoter binding factor 1) transcriptional activity (Kopan and Ilagan 2009). However, the mechanisms for aberrant activation of the Notch signaling pathway in cancers have not been fully elucidated. Recent studies have indicated that the Notch signaling pathway may play instrumental roles in cancer stem cells. Notch signaling activation suppresses differentiation and enhances self-renewal and survival of neural progenitor cells (Yoon and Gaiano 2005). Suppression of Notch signaling activation eradicates the stem-like population in embryonal brain tumors and reduces xenograft formation (Fan et al. 2006). In breast cancer, inhibition of Notch signaling activation by γ-secretase inhibitor reduces sphere-forming ability (Farnie and Clarke 2007). While the mechanisms of Notch oncogenic functions have been well documented, the mechanism of Notch in cancer stem cells is just emerging.

NDR1/STK38 kinase is a member of evolutionarily conserved nuclear-Dbf2-related (NDR) serine-threonine protein kinase family, which play important roles in multiple biological processes, such as centrosome duplication, cell proliferation, apoptosis, and neuron development (Hergovich et al. 2006). The role of NDR1 in cancers is complicated. NDR1 might function as a tumor suppressor to suppress metastasis by inactivating epithelial-mesenchymal transition in prostate cancer cells (Yue, et al. 2018). On the contrary, NDR1 might also act as an oncogene to stabilize MYC protein in a kinase activity-dependent manner and support the growth of B-cell lymphoma (Bisikirska et al. 2013). Recent studies suggested that NDR1 might participate in the regulation of stem cell function. Downregulation of NDR1 increases the numbers of long-term culture initiating cells (Ali et al. 2009). Spindle orientation is critical for a wide range of developmental processes, including stem cell division (Bergstralh et al. 2017). Abnormal NDR1 activation disrupts mitotic spindle orientation (Yan et al. 2015). Hippo pathway is a tumor suppressor pathway with a profound impact on stem cells (Park et al. 2018). NDR1 kinases can act as a YAP kinases to modulate the activity of the Hippo pathway (Hergovich 2016). Currently, the roles of NDR1 in cancer stem cells and the underlying regulatory mechanisms remain unclear.

Altogether, these observations encouraged us to investigate the roles of NDR1 in BCSCs and the underlying regulatory mechanisms. We also sought to determine whether NDR1 and Notch pathway might have crosstalk, and how this potential crosstalk participates in the regulation of BCSC properties.

## Materials and methods

### Reagents and cell culture

Taxol, Cycloheximide (CHX), and MG132 were purchased from Sigma-Aldrich. Epirubicin was purchased from Selleck. Antibodies against Caspase-3, Caspase-8, Caspase-9, PARP, Notch1, NICD, c-myc, HES1, Flag, HA, myc, ADAM10/17, Presenilin 1/2, pStat3(Y705), Stat3, pNF‐κB, NF‐κB, and GAPDH were purchased from Cell Signaling Technology. Antibody against NDR1 was purchased from Santa Cruz Biotechnology. Antibody against NDR1 (Phospho-Thr444/442) was purchased from SABbiotech. MCF-7 cells were obtained from the ATCC and were cultured in DMEM (Thermo Fisher Scientific) supplemented with 10% fetal bovine serum (Thermo Fisher Scientific). SUM149 cell line was obtained from Asterand and was cultured in Ham's F-12 supplemented with 5% fetal bovine serum. Cells were maintained at 37℃ in a humidified 5% CO2 atmosphere.

### Apoptosis analysis

Annexin V-FITC Apoptosis Detection Kit was purchased from Sigma-Aldrich. Cells were collected at the indicated time point. Cells were resuspended in binding buffer (500 μl/sample). Cells were then stained with Annexin-V-FITC (5 μl/sample). Finally, cells were stained with PI (1 μl/sample). After the above incubation, cells were subjected to flow cytometry detection (BD Biosciences).

### Western blot analysis

Proteins were resolved by SDS-PAGE. 15 μg of total protein were resolved and transferred to nitrocellulose membranes. Nitrocellulose membranes were blocked for 1 h in blocking buffer (4.0% bovine serum albumin [BSA], 10 mM PBS, 0.05% Triton X-100, pH 7.4) at 25° C. Membranes were next incubated with the primary antibody in blocking buffers overnight at 4° C with gentle agitation. Membranes were washed 3 times in 10 mM PBS, 0.05% Triton X-100, pH 7.4, and then incubated in secondary antibody at room temperature for 1 h. Membranes were washed 3 times in 10 mM PBS, 0.05% Triton X-100 pH 7.4; and protein products were evaluated with an enhanced chemiluminescence kit (Pierce). In the densitometry analysis, the relative pixel density of the target protein was normalized to that of GAPDH.

### CD24/44 analysis

CD24-PE and CD44-FITC were purchased from Thermo Fisher Scientific. Cells were harvested and resuspended in cold PBS at a concentration of 1 × 10^6^ cells per 100 μl. Cells (100 μl) were incubated with 5 μl antibody on ice for 30 min. Then, cells were subjected to flow cytometric analysis.

### ALDEFLUOR assay

ALDEFLUOR assay kit was purchased from Stem Cell Technologies. Cells were incubated with the ALDEFLUOR solution, which supplemented with or without N,N-diethylaminobenzaldehyde (DEAB), at 37˚C for 45 min. Cells were analyzed using flow cytometry.

### Sphere formation assay

The sphere formation assay was performed as previously described (Zheng et al. 2016). Single cells were plated in ultra-low attachment 6-well plates at a density of 500 viable cells/ml. Cells were cultured in sphere culture medium for 7 days. Spheres were photographed using an inverted microscope (100 × , Olympus). The diameters of spheres were measured by Image pro plus 6.0 software (Media Cybernetics).

### Luciferase reporter assay

Luciferase activity was detected by using the Dual-Luciferase Reporter Assay Kit (Promega) according to the manufacturer's instructions. Cells were lysed with passive lysis buffer. Ten microlitres of lysate were sequentially subjected to Firefly or Renilla luciferase activity. Transcriptional activity was calculated as the ratio of firefly luciferase activity to Renilla luciferase activity.

### Real-time PCR

Reverse transcription was performed with the M-MLV Reverse Transcriptase (Promega). Real-time PCR was carried out using an ABI PRISM 7500 Sequence Detection System (Applied Biosystems). Reactions were run in triplicate in three independent experiments. The geometric mean of housekeeping gene GAPDH was used as an internal control to normalize the variability in expression levels. The primer sequences are provided as follows: 1. C-MYC, 5′-GCC ACG TCT CCA CAC ATC AG and 5′-TGG TGC ATT TTC GGT TGT TG; 2. HES1, 5′-AAG AAA GAT AGC TCG CGG CA and 5′-TAC TTC CCC AGC ACA CTT GG; 3. GAPDH, 5′-TGC ACC ACC AAC TGC TTA GC and 5′-GGC ATG GAC TGT GGT CAT GAG.

### Immunoprecipitation

Immunoprecipitation was performed as previously described (Zheng et al. 2016).

### Ubiquitination analysis

Cells were transfected with indicated plasmids. Cells were lysed in RIPA supplemented with protease and phosphatase inhibitors. Total His‐tagged proteins purified on nickel‐NTA–agarose. Total proteins and His‐tagged proteins were then resolved by SDS–PAGE and detected by immunoblotting.

### RNA interference

The target sequences of NDR1 siRNA (GenePharma) were as follows: 5′-GTATTAGCCATAGACTCTATT. The shRNA against NDR1 were designed according to siRNA and cloned into pLKO.1-puro (Addgene). The target sequences of NOTCH1 shRNA was as follows: 5′-CCGGGACATCACGGATCATAT, and was cloned into pLKO.1-puro.

### Lentivirus preparation and transfection

293 T (5 × 10^6^) cells were transfected with 24 μg plasmids (lentiviral vector:psPAX2:pMD2.G = 4:3:1). The virus particles containing supernatant were harvested at 24 h and 48 h. The multiplicity of infection for transfection was 5.

### Kaplan–meier plotter database

The Kaplan–Meier plotter database (http://kmplot.com) allows the acquirement of the effect of 30 k genes on survival in 25 k + samples from 21 tumor types, especially for breast cancer (Gyorffy et al. 2010). Survival analysis was performed by Kaplan–Meier plotter database. The mRNA expression of NDR1 from gene chip data was selected for survival analysis. The Affy ID of NDR1was 202951. Breast cancer patients were split into high-NDR1 expression group and low-NDR1 expression group by the function of auto select best cutoff. For OS, RFS, PPS, and DMFS, the optimal cutoff values were 1444, 994, 1606 and 1638, respectively. The follow up threshold was 120 months. The relationship of NDR1 mRNA expression and survival [OS (n = 1880), RFS (n = 4934), PPS (n = 458), or DMFS (n = 2767)] was obtained with the calculation of hazard ratio with 95% confidence intervals, and log-rank P value.

### Statistical analysis

The statistical analysis was performed using SPSS version 16.0 (SPSS Inc.). The Kaplan–Meier analysis and log-rank test were used to evaluate survival. The correlation between NDR1 and NICD was determined by Spearman correlation test. Statistical analysis of sphere size was evaluated by the Kruskal–Wallis test followed by Dunn's multiple comparison test. The ANOVA test, followed by the Least Significant Difference test, was used when performing multiple comparisons. The unpaired Student's t-test was used to compare two groups. The level of significance was set at P < 0.05.

## Results

### NDR1 modulates apoptosis and drug resistance in breast cancer cells.

To evaluate the biological function of NDR1 in breast cancer cells, we first examined whether NDR1 affected proliferation in breast cancer cells. As shown in Additional file 1: Fig. S1a, NDR1 was expressed at low level in MCF-10A, moderate level in MCF-7 and SUM149, and high level in MDA-MB-231 and BT549. We chose MCF-7 and SUM149 cells for the following study. Overexpression of NDR1 did not promote the proliferation of SUM149 and MCF-7 cells under traditional culture condition (Additional file 1: Fig. S1b–d). Downregulation of NDR1 did not decrease the proliferation of SUM149 and MCF-7 cells under traditional culture condition (Additional file 1: Fig. S1e–g).

**Fig. 1:**
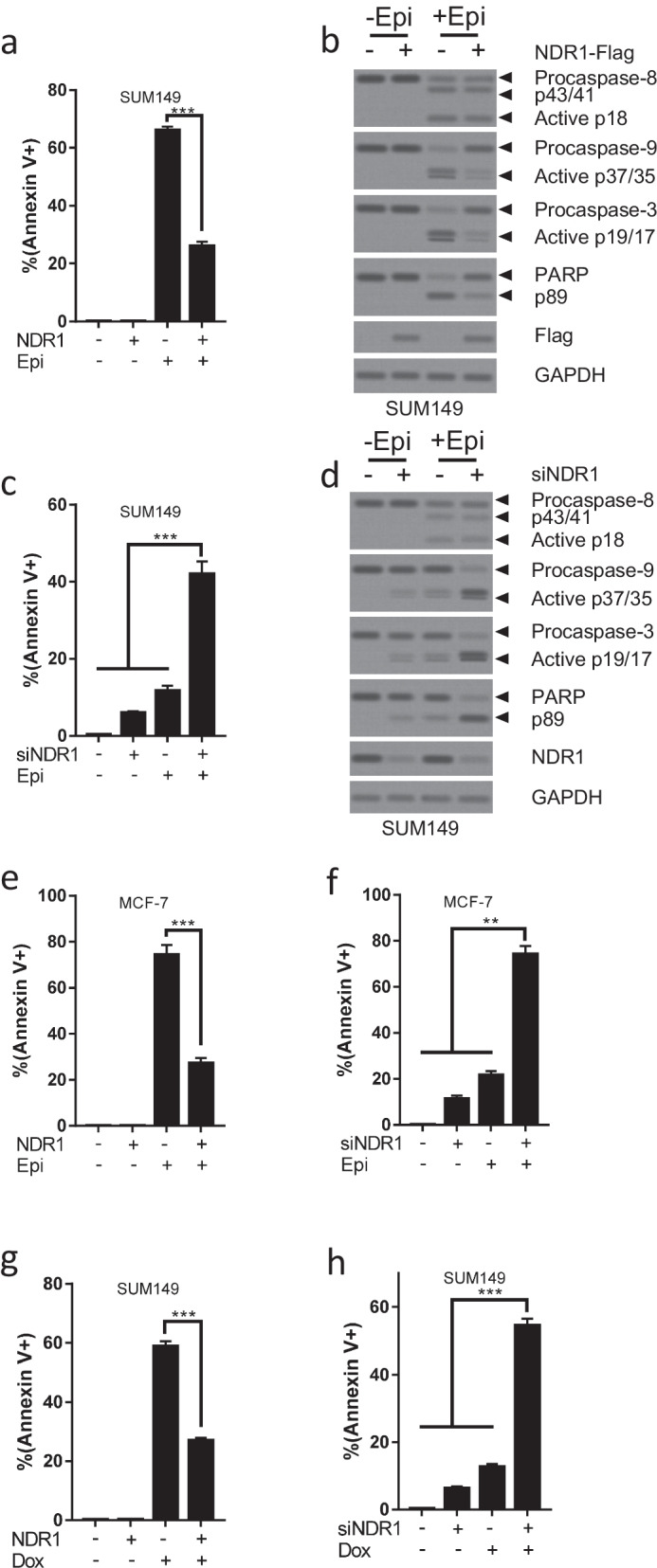
NDR1 modulates apoptosis and drug sensitivity in breast cancer cells. **a**, **b** NDR1 or control vector was expressed in SUM149 cells for 24 h. Then, SUM149 cells were treated with or without Epirubicin (2.5 µM) for 48 h. Cells were harvested and subjected to Annexin V-FITC staining, flow cytometry analysis and western blot analysis. **c**, **d** NDR1 siRNA or control siRNA were transfected in SUM149 cells for 24 h. Then, SUM149 cells were treated with or without Epirubicin (0.5 µM) for 48 h. Cells were harvested and subjected to Annexin V-FITC staining, flow cytometry analysis and western blot analysis. **e** NDR1 or control vector were expressed in MCF-7 cells for 24 h. Then, MCF-7 cells were treated with or without Epirubicin (2.5 µM) for 48 h. Cells were harvested and subjected to Annexin V-FITC staining and flow cytometry analysis. **f** NDR1 siRNA or control siRNA were transfected in MCF-7 cells for 24 h. Then, MCF-7 cells were treated with or without Epirubicin (0.5 µM) for 48 h. Cells were harvested and subjected to Annexin V-FITC staining and flow cytometry analysis. **g** NDR1 or control vector were expressed in SUM149 cells for 24 h. Then, SUM149 cells were treated with or without Taxol (20 nM) for 48 h. Cells were harvested and subjected to Annexin V-FITC staining and flow cytometry analysis. **h** NDR1 siRNA or control siRNA were transfected in SUM149 cells for 24 h. Then, SUM149 cells were treated with or without Taxol (5 nM) for 48 h. Cells were harvested and subjected to Annexin V-FITC staining and flow cytometry analysis. The bar represents mean ± SD of three independent experiments (*: p < 0.05, **: p < 0.01, ***: p < 0.001)

We next examined whether NDR1 regulated apoptosis in breast cancer cells. Overexpression of NDR1 did not induce apoptosis and activate the apoptosis pathway in SUM149 cells (Fig. 1a, b, and Additional file 1: Fig. S1h), while downregulation of NDR1 induced apoptosis and activate apoptosis pathway in SUM149 cells (Fig. 1c, d, and Additional file 1: Fig. S1i). Similarly, these phenomena were also observed in MCF-7 cells (Fig. 1e, f, and Additional file 1: Fig. S1j, k). We further evaluated whether NDR1 regulated drug sensitivity. We first determined the apoptosis of SUM149 or MCF-7 cells treated with indcated concentration of Epirubicin (Epi) (Additional file 1: Fig. S1l, m). The concentration of 0.5 and 2.5 µM Epi were chosen for RNAi assay and overexpression assay respectively. As shown in Fig. 1a and Additional file 1: Fig. S1h, treatment with Epi induced significant apoptosis in SUM149, while overexpression of NDR1 markedly decreased Epi-induced apoptosis. Consistently, overexpression of NDR1 also reversed Epi-induced activation of the apoptotic pathway (Fig. 1b). Moreover, the downregulation of NDR1 significantly enhanced Epi-induced apoptosis and activation of the apoptotic pathway (Fig. 1c, d, and Additional file 1: Fig. S1i). These data indicated that NDR1 was critical for regulating drug sensitivity. We further confirmed that the expression of NDR1 also affected the drug sensitivity of Taxol (Tax) in SUM149 cells (Fig. 1g, h, and Additional file 1: Fig. S1n, o).

### NDR1 is critical for the regulation of cancer stem cell properties in breast cancer cells

**Fig. 2:**
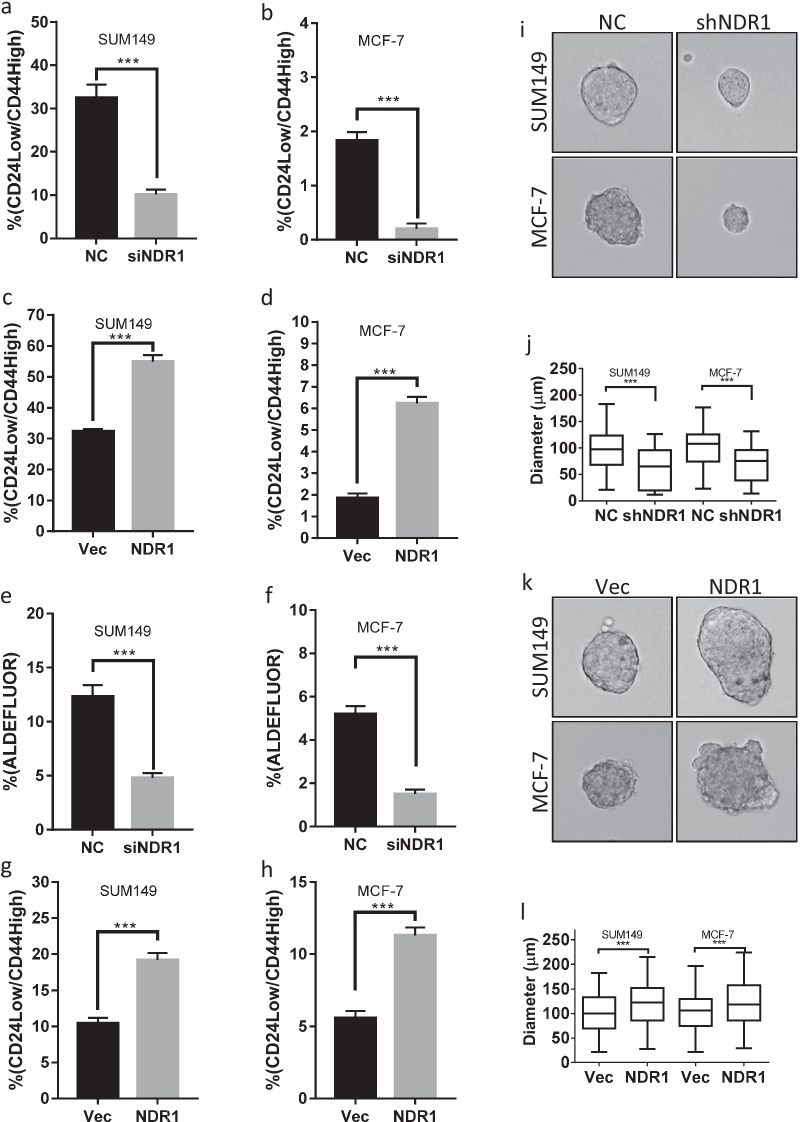
NDR1 is critical for the regulation of cancer stem cell properties in breast cancer cells. **a**, **b** NDR1 siRNA or control siRNA were transfected in SUM149 or MCF-7 cells for 72 h. Cells were harvested and subjected to CD24/44 staining and flow cytometry analysis. **c**, **d** NDR1 or control vector were expressed in SUM149 or MCF-7 cells for 72 h. Cells were harvested and subjected to CD24/44 staining and flow cytometry analysis. **e**, **f** NDR1 siRNA or control siRNA were transfected in SUM149 or MCF-7 cells for 72 h. Cells were harvested and subjected to ALDEFLUOR staining and flow cytometry analysis. **g**, **h** NDR1 or control vector were expressed in SUM149 or MCF-7 cells for 72 h. Cells were harvested and subjected to ALDEFLUOR staining and flow cytometry analysis. **i**, **j** SUM149 or MCF-7 cells expressed NDR1 shRNA or control vector were subjected to sphere-forming assay. The representative spheres were shown in **i**. The distribution of sphere diameters was shown in **j**. **k**, **l** SUM149 or MCF-7 cells expressed NDR1 or control vector were subjected to sphere-forming assay. The representative spheres were shown in k. The distribution of sphere diameters was shown in l. The bar represents mean ± SD of three independent experiments (*: p < 0.05, **: p < 0.01, ***: p < 0.001)

Cancer stem cells are an important population responsible for drug resistance (Dean et al. 2005). The previous study showed that NDR1 might be important for the maintenance of normal stem cells (Ali et al. 2009). We evaluated whether NDR1 might participate in the regulation of breast cancer stem cells (BCSCs). As shown in Fig. 2a, b and Additional file 1: Fig. S2a, b, inhibition of NDR1 expression reduced CD24^low^/CD44^high^ population in SUM149 and MCF-7 cells. On the contrary, the upregulation of NDR1 expression increased CD24^low^/CD44^high^ population in SUM149 and MCF-7 cells (Fig. 2c, d and Additional file 1: Fig. S2c, d). The activity of aldehyde dehydrogenase (ALDH) is a hallmark of cancer stem cells (Marcato et al. 2011). We confirmed the effect of NDR1 on BCSCs by measuring the population of high ALDH activity. Our data showed that inhibition of NDR1 expression also reduced ALDEFLUOR^high^ population in SUM149 and MCF-7 cells (Fig. 2e, f and Additional file 1: Fig. S2e, f), while the upregulation of NDR1 expression increased ALDEFLUOR^high^ population in SUM149 and MCF-7 cells (Fig. 2g, h and Additional file 1: Fig. S2g, h). Since kinase activity is critical for NDR1 biological functions, we test whether kinase activity might important for regulation of BCSCs properties. Mutation at K118A of NDR1, a kinase-dead (KD) mutation in the catalytic site of NDR1 (Ma et al. 2017), impaired NDR1 activation as indicated by Thr444/442 phosphorylation (Additional file 1: Fig. S2i). Our data showed that both wild type or kinase dead NDR1 increased CD24^low^/CD44^high^ population in SUM149 cells at a similar level (Additional file 1: Fig. S2j), indicating that NDR1 might regulate BCSCs properties by a kinase activity-independent mechanism. Besides, sphere-forming assays were used to characterize in vitro cancer stem cell properties. Suppression of NDR1 expression significantly inhibited the sphere-forming ability of SUM149 and MCF-7 cells (Fig. 2i, j), while overexpression of NDR1 significantly enhanced the sphere-forming ability of SUM149 and MCF-7 cells (Fig. 2k, l). Thus, our data suggested that NDR1 could be an essential factor to control BCSCs properties.

Endocrine therapy is a very effective strategy to decrease proliferation of estrogen receptor (ER) positive breast cancer cells (Jordan and Brodie 2007). We further evaluated whether NDR1 might invole in ER-related response. We found that after estrogen stimulation, the proportion of CD24^low^/CD44^high^ population in estradiol group was significantly higher than that in vehicle control in MCF-7 cells (Additional file 1: Fig. S3a), whereas no significant change in the proportion of CD24^low^/CD44^high^ population was observed when the same treatment conditions were imposed on SUM149 cells, which lacked ER expression (Additional file 1: Fig. S3a). However, estrogen stimulation did not significantly affect the protein expression of NDR1 in both SUM149 and MCF-7 cells (Additional file 1: Fig. S3b), indicating that NDR1 might not regulate ER stimulated BCSCs properties. Tamoxifen, the potent estrogen antagonist, suppressed proliferation in MCF-7 cells, but not in SUM149 cells which lacked ER expression (Additional file 1: Fig. S3c, d). Overexpression of NDR1 did not reverse the inhibitory effect induce by Tamoxifen in MCF-7 cells (Additional file 1: Fig. S3c).

### NDR1 increases Notch1 signaling activity in breast cancer cells

**Fig. 3:**
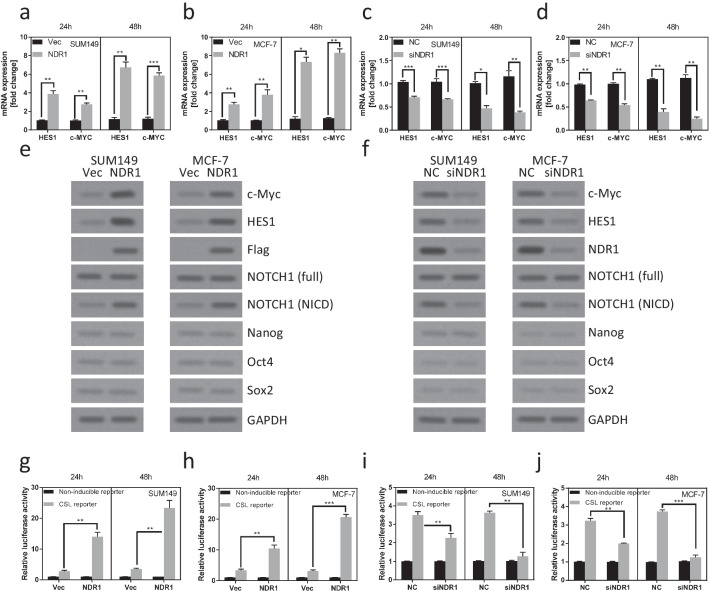
NDR1 increases Notch1 signaling activity in breast cancer cells. **a**, **b** NDR1 or control vector were expressed in SUM149 or MCF-7 cells for 48 h. Cells were harvested and subjected to Real-time PCR analysis. **c**, **d** NDR1 siRNA or control siRNA were transfected in SUM149 or MCF-7 cells for 48 h. Cells were harvested and subjected to Real-time PCR analysis. **e** NDR1 or control vector were expressed in SUM149 or MCF-7 cells for 48 h. Cells were harvested and subjected to western blot analysis. **f** NDR1 siRNA or control siRNA were transfected in SUM149 or MCF-7 cells for 48 h. Cells were harvested and subjected to western blot analysis. **g**, **h** Luciferase reporter (Notch1 or control) and Renilla reporter were transfected into SUM149 or MCF-7 cells for 12 h. Then, NDR1 or control vector was expressed in SUM149 or MCF-7 cells for 48 h. Cells were harvested and subjected to luciferase reporter assay. **i**, **j** Luciferase reporter (Notch1 or control) and Renilla reporter were transfected into SUM149 or MCF-7 cells for 12 h. Then, NDR1 siRNA or control siRNA were transfected in SUM149 or MCF-7 cells for 48 h. Cells were harvested and subjected to luciferase reporter assay. The bar represents mean ± SD of three independent experiments (*: p < 0.05, **: p < 0.01, ***: p < 0.001)

We next demonstrated the mechanism of NDR1 regulated BCSCs properties. We noticed that some downstream targets, such as HES-1 (Moriyama et al. 2006) and c-MYC (Palomero et al. 2006), of the Notch1 pathway were upregulated in SUM149 and MCF-7 cells when NDR1 was overexpressed (Fig. 3a, b). In contrast, downregulation of NDR1 expression decreased the mRNA expression of HES-1 and c-MYC in SUM149 and MCF-7 cells (Fig. 3c, d). Consistently, the expression of HES-1 and c-MYC protein was regulated by NDR1 in SUM149 and MCF-7 cells (Fig. 3e, f). The other self-renewal transcription factors, such as Nanog, Oct4 or Sox2, was not regulated by NDR1 in SUM149 and MCF-7 cells (Fig. 3e, f). During Notch1 signaling activation, Notch1 undergoes two steps cleavage to release the Notch intracellular domain (NICD), which translocates into the nucleus to modulate the expression of downstream targets (Bray 2016). We first detected the basal expression of Notch1 and NICD in several breast cell lines (Additional file 1: Fig. S1a). Interestingly, we observed that the expression of NICD was also affected by NDR1 expression, while the full-length Notch1 protein was not affected (Fig. 3e, f). To further confirm NDR1 regulated Notch1 signaling activation, we examined whether NDR1 regulated Notch reporter activation. As shown in Fig. 3g, h, the upregulation of NDR1 expression significantly enhanced Notch reporter activation. Downregulation of NDR1 expression significantly suppressed Notch reporter activation (Fig. 3i, j). These data implied that Notch1 signaling could be one of the most important pathways mediating NDR1 regulated BCSCs properties.

### Activation of Notch1 signaling pathway is essential for NDR1 enhanced cancer stem cell properties in breast cancer cells

**Fig. 4:**
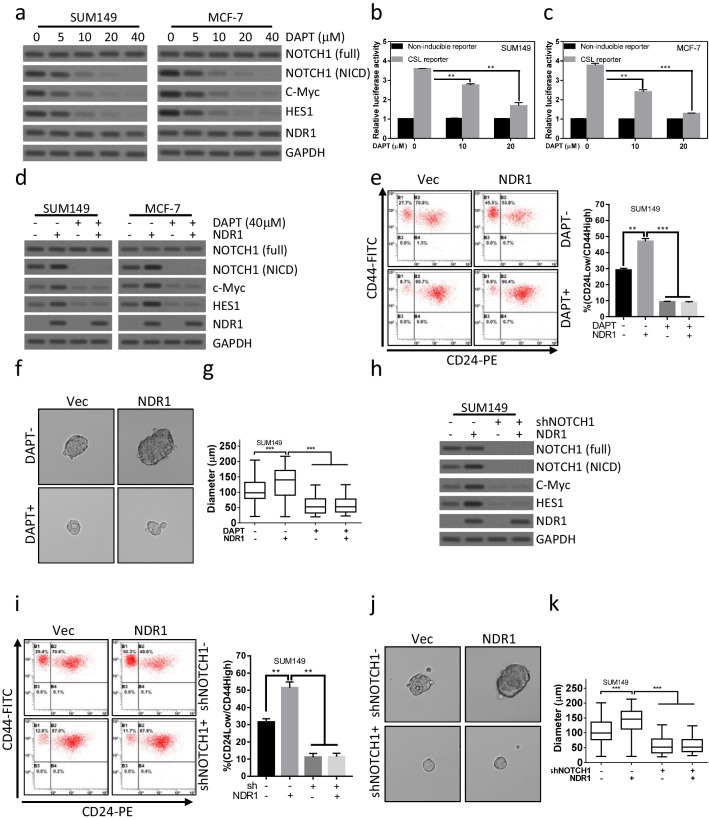
Activation of Notch1 signaling pathway is essential for NDR1 enhanced cancer stem cell properties in breast cancer cells. **a** SUM149 or MCF-7 cells were treated with the indicated concentration of DAPT for 72 h. Cells were harvested and subjected to western blot analysis. **b**, **c** SUM149 or MCF-7 cells were treated with the indicated concentration of DAPT for 72 h. Cells were harvested and subjected to luciferase reporter assay. **d** NDR1 or control vector were expressed in SUM149 or MCF-7 cells. Cells were treated with the indicated concentration of DAPT for 72 h. Cells were harvested and subjected to western blot analysis. **e–g** NDR1 or control vector was expressed in SUM149 cells. Cells were treated with the indicated concentration of DAPT for the indicated time. Cells were harvested and subjected to CD24/44 analysis (72 h, **e**) and sphere-forming assay (7 days, **f** and **g**). The representative spheres were shown in **f**. The distribution of sphere diameters was shown in g. **h** NDR1 or control vector was expressed in SUM149 cells. Cells were infected with lentivirus expressed shNotch1 or control shRNA for the indicated time. Cells were harvested and subjected to western blot analysis. **i-k** NDR1 or control vector was expressed in SUM149 cells. Cells were infected with lentivirus expressed shNotch1 or control shRNA for the indicated time. Cells were harvested and subjected to CD24/44 analysis (72 h, **i**) and sphere-forming assay (7 days, **j** and **k**). The representative spheres were shown in j. The distribution of sphere diameters was shown in k. The bar represents mean ± SD of three independent experiments (*: p < 0.05, **: p < 0.01, ***: p < 0.001)

We evaluated the critical role of Notch1 signaling in NDR1 regulated BCSC properties. Notch pathway inhibition using DAPT, a γ-secretase inhibitor (GSI), prevented proteolytic cleavage of NICD (Fig. 4a) and transcription activation (Fig. 4b, c), thereby reducing the expression of HES-1 and c-MYC (Fig. 4a). As shown in Fig. 4d, blocking proteolytic cleavage of NICD by DAPT abolished NDR1 induced HES-1 and c-MYC expression. Interestingly, overexpression of NDR1 failed to increase CD24^low^/CD44^high^ population (Fig. 4e) and enhance sphere-forming ability in SUM149 (Fig. 4f, g) and MCF-7 (Additional file 1: Fig. S4a, b) cells when NICD cleavage was blocked by DAPT. To more specific study Notch1 function in NDR1 regulated BCSCs properties, Notch1 expression was downregulated by shRNA (Fig. 4h). NDR1 induced upregulation of HES-1 and c-MYC was abolished when Notch1 expression was suppressed (Fig. 4h). As shown in Fig. 4i, NDR1 overexpression failed to increase CD24^low^/CD44^high^ population when Notch1 expression was downregulated. Consistently, NDR1 enhanced sphere-forming ability was abolished by inhibiting Notch1 expression (Fig. 4j, k). Thus, these data suggested that Notch1 could be a critical regulator mediated NDR1 enhanced BCSC properties.

### NDR1 suppresses FBW7 mediated degradation of NICD in breast cancer cells

**Fig. 5:**
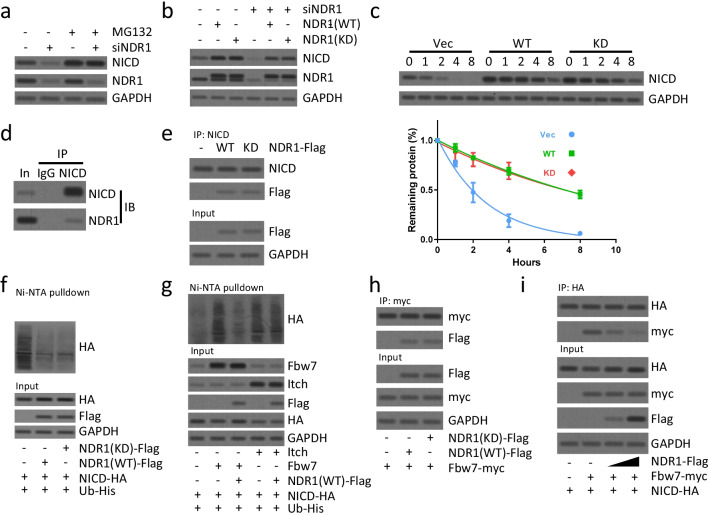
NDR1 suppresses FBW7 mediated degradation of NICD in breast cancer cells. **a** NDR1 siRNA or control siRNA were transfected in SUM149 cells for 48 h. Then, cells were treated with MG132 (10 μM) for 2 h. Cells were harvested and subjected to western blot analysis. **b** NDR1 siRNA or control siRNA were transfected in SUM149 cells for 48 h. Then, wild type or kinase-dead (K118A) NDR1 was overexpressed for 24 h. Cells were harvested and subjected to western blot analysis. **c** Wild type, kinase-dead NDR1 or control vector was overexpressed in SUM149 cells for 24 h. Cells were treated with CHX (50 µg/ml) for the indicated time. Cells were harvested and subjected to western blot analysis. The densitometric quantification of NICD normalized to GAPDH was plotted against various time points to determine the half-life of NICD. **d** The total protein of SUM149 cells was subjected to immunoprecipitation and western blot analysis using antibodies as indicated. **e** Wild type, kinase-dead NDR1 or control vector was overexpressed in SUM149 cells for 24 h. The total protein of SUM149 cells was subjected to immunoprecipitation and western blot analysis using antibodies as indicated. **f**, **g** SUM149 cells were transfected with indicated vectors for 24 h. Cells were subsequently treated with MG132 (10 μM) for 2 h prior to ubiquitination proteins were pulldown with Ni–NTA beads and followed by western blot analysis for indicated proteins. **h**, **i** SUM149 cells were transfected with indicated vectors for 24 h. The total protein of SUM149 cells was subjected to immunoprecipitation and western blot analysis using antibodies as indicated

We next determined how NDR1 modulated Notch1 signaling activity. Since we did not observe significant changes in NOTCH1 (full length) protein when NDR1 was upregulated or downregulated (Fig. 3e, f), we considered that NDR1 was less likely to modulate transcription or translation of NOTCH1. Activation of Notch signaling is controlled by ligand binding, membrane-proximal cleavage by a metalloprotease, transmembrane domain cleavage by γ-secretase, releasing NICD to enter the nucleus and transcription of downstream target genes (Bray 2016). Neither ADAM10/17 expression (Christian 2012) nor Presenilin 1/2 expression (Shih Ie and Wang 2007) was affected by the upregulation or downregulation of NDR1 (Additional file 1: Fig. S5), indicating that the upregulation of NICD was not caused by NOTCH1 receptor cleavage. We further tested whether NDR1 might modulate NICD expression via proteasome degradation mechanism. Suppression of NICD induced by downregulation of NDR1 was reversed by MG132 treatment (Fig. 5a). NDR1 kinase activity played a critical role during executing its biological function (Tamaskovic et al. 2003). Interestingly, our data showed that both wild type (WT) and kinase dead (KD) NDR1 rescued NICD expression when endogenous NDR1 was suppressed (Fig. 5b), indicating a kinase independent function of NDR1. Consistently, cycloheximide chase assay showed that both WT and KD NDR1 stabilized NICD at a similar level (Fig. 5c).

We further examined how NDR1 stabilized NICD. Co-immunoprecipitation (Co-IP) assay indicated that endogenous NDR1 interacted with endogenous NICD (Fig. 5d). The loss of NDR1 kinase activity did not affect the interaction between NDR1 and NICD (Fig. 5e). We tested whether NDR expression affected NICD ubiquitination. Indeed, we observed that the stabilizing effect of NDR1 overexpression on NICD was due to impaired NICD ubiquitination (Fig. 5f). Fbw7/Sel10 regulates PEST-dependent NICD turnover in the nucleus (Oberg et al. 2001), and Itch regulates the PEST-independent turnover of cytoplasmic Notch proteins (Qiu et al. 2000). We tested the effect of NDR1 overexpression on FBW7- or Itch-mediated ubiquitination. Although the ubiquitination of NICD by Itch was not impaired by NDR1 overexpression (Fig. 5g), the ubiquitination of NICD by Fbw7 was impaired by NDR1 overexpression (Fig. 5g). Interestingly, NDR1 also interacted with Fbw7 in a kinase activity-independent manner (Fig. 5h). Importantly, NDR1 competed with Fbw7 for NICD interaction (Fig. 5i). Collectively, our data implied that NDR1 might regulate NICD protein stability by interfering with Fbw7-mediated ubiquitination in a kinase activity-independent manner.

### NDR1 mediates IL-6, TNF-α or Wnt3a induced activation of Notch1 signaling pathway and enrichment of BCSCs

**Fig. 6:**
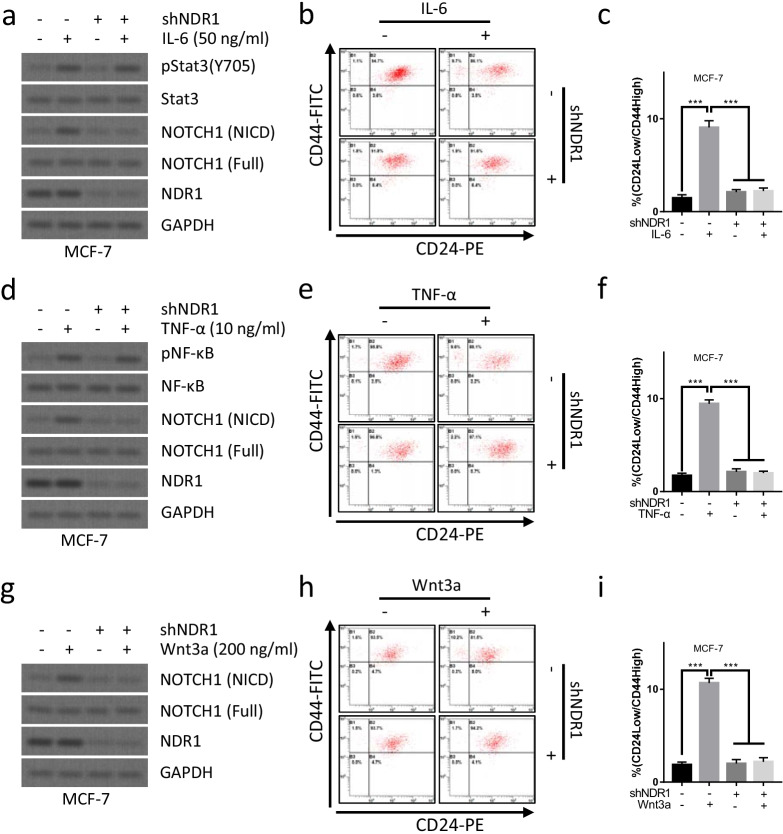
NDR1 mediates IL-6, TNF-α or Wnt3a induced activation of Notch1 signaling pathway and enrichment of breast cancer stem cells**. a**–**c** MCF-7 cells were treated with or without IL-6 or shNDR1 for 7 days. The cell were collected for western blot analysis or CD24/44 analysis. **d**–**f** MCF-7 cells were treated with or without TNF-α or shNDR1 for 7 days. The cell were collected for western blot analysis or CD24/44 analysis. **g**–**i** MCF-7 cells were treated with or without Wnt3a or shNDR1 for 7 days. The cell were collected for western blot analysis or CD24/44 analysis. The bar represents mean ± SD of three independent experiments (*: p < 0.05, **: p < 0.01, ***: p < 0.001)

Since multiple signaling pathways, such as IL-6/JAK2/STAT3 pathway (Song, et al. 2020), TNF-α/NF-κB pathway (Ando et al. 2003) or Wnt/β-catenin pathway (Wang et al. 2016), might crosstalk with and activate Notch1 signaling pathway. We examined whether NDR1 might essential for the activation of Notch1 signaling pathway induced by IL-6/JAK2/STAT3 pathway, TNF-α/NF-κB pathway or Wnt/β-catenin pathway. As shown in Fig. 6a, the activation of IL-6/JAK2/STAT3 pathway enhanced the expression of NICD protein, indicating the activation of Notch1 signaling pathway. Downregulation of NDR1 expression abolished the the activation of Notch1 signaling pathway induced by IL-6 treatment (Fig. 6a), indicating that NDR1 was critical for IL-6/JAK2/STAT3 induced activation of Notch1 signaling pathway. Interestingly, downregulation of NDR1 expression also abolished IL-6 treatment induced enrichment of CD24^low^/CD44^high^ population in MCF-7 cells (Fig. 6b, c), indicating that NDR1 was also essential for IL-6/JAK2/STAT3 induced enrichment of BCSCs. We further performed similar experiment to evaluate the role of NDR1 in the regulation of TNF-α/NF-κB pathway or Wnt/β-catenin pathway induced activation of Notch1 signaling pathway. Similary, TNF-α (Fig. 6d) or Wnt3a (Fig. 6g) failed to enhance the activation of Notch1 signaling pathway when NDR1 expression was suppressed. Downregulation of NDR1 expression also abolished TNF-α (Fig. 6e, f) or Wnt3a (Fig. 6h, i) treatment induced enrichment of CD24^low^/CD44^high^ population in MCF-7 cells. These data indicated that NDR1 might function as a pivot to modulate the activation of Notch1 signaling pathway by multiple signaling pathways, NDR1 might also essential for IL-6/JAK2/STAT3 pathway, TNF-α/NF-κB pathway or Wnt/β-catenin pathway induced enrichment of BCSCs by activating Notch1 signaling pathway.

### The clinical relevance of NDR1

**Fig. 7:**
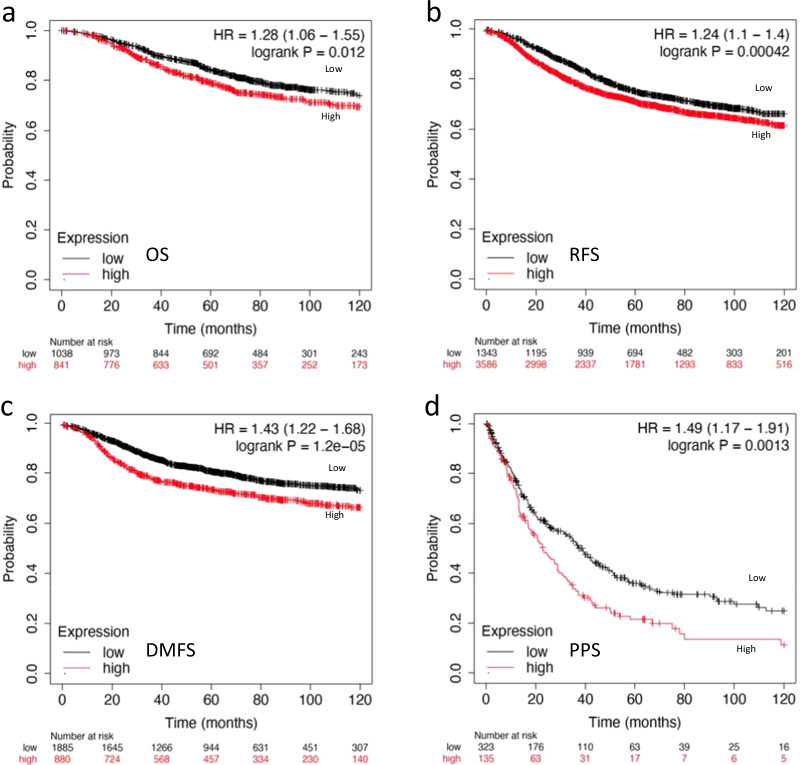
The clinical relevance of NDR1. **a**–**d** Kaplan–Meier analysis and the log-rank test were performed to evaluate the correlation between NDR1 expression and OS/RFS/DMFS/PPS

Due to the critical role of NDR1 in regulating NICD expression and BCSC properties, we examined the correlation between survival and NDR1 expression. The survival analysis was accomplished by Kaplan–Meier plotter database (http://kmplot.com). The mRNA expression of NDR1 (ID: 202951) from gene chip data was used for survival analysis. The cut-off point considered to differentiate between high and low expression of NDR1 was determined by cut-off plots (Fig. S6a-S6d). A higher NDR1 expression indicated a poorer prognosis for patients as indicated by overall survival (OS, n = 1880) (Fig. 7a). A higher NDR1 expression also predicted an inferior relapse free survival (RFS, n = 4934), distant metastasis free survival (DMFS, n = 2767) or post progression survival (PPS, n = 458) (Fig. 7b–d). These data indicated that NDR1 expression could be a prognostic predicting marker in breast cancer.

## Discussion

The Hippo pathway is a crucial regulator of development through controlling cellular functions such as cell proliferation, apoptosis, differentiation and stemness (Pan 2010). The NDR family members, an evolutionarily conserved subclass of the AGC group of kinases, are considered as novel members of the Hippo core cassette (Hergovich 2016). NDR kinase might function in concert with the Hippo pathway to play critical roles in the regulation of cell proliferation, apoptosis, differentiation and stemness (Hergovich 2016). Notch pathway is an evolutionarily conserved pathway to regulate cell proliferation, apoptosis, cell fate, and differentiation in many cell types and at various stages during development (Andersson et al. 2011). Although Hippo and Notch pathways are often defined as the different pathways that influence cell and organismal fate, they might function as networks that crosstalk. In the current study, we showed that NDR1 might participate in Notch pathway regulation by increasing NICD protein stability. Our data might suggest a potential mechanism to explain the interaction between Hippo and Notch pathways. Interestingly, this mechanism might be critical for the regulation of BCSC properties.

The crosstalk between Hippo and Notch pathways might happen at other levels. YAP1, a rate-limiting transcription factor in the Hippo pathway (Huang et al. 2005), could be a downstream target of Notch1 in neural stem cells (Li et al. 2012). Reciprocally, YAP1 transcriptionally upregulates the expression of Notch pathway molecules, including Notch ligand JAG1 and DLL1 and the core Notch transcription factor RBPJ (Tschaharganeh et al. 2013; Slemmons et al. 2017). These studies imply that the components of Hippo and Notch pathways might form a positive feedback loop which is essential to maintaining the high Notch signaling activity in cancer cells. Interestingly, MST1/2, upstream suppressive kinases of the transcriptional coactivators YAP/TAZ (Meng et al. 2016), might act as critical gatekeepers to keep the positive feedback loop in check in normal cells (Kim et al. 2017). Besides, loss of Nf2, a Hippo pathway tumor suppressor, results in increased expression of Notch2 in cholangiocytes (Wu et al. 2017). Moreover, the Hippo pathway might regulate endocytic trafficking of Notch receptor to control follicle-cell differentiation and oocyte-polarity formation (Yu et al. 2008). Our study suggested that proteasome degradation mechanism mediated the crosstalk between Hippo and Notch pathways.

The activation of the Notch pathway is strictly controlled through ligand binding, proteolytic cleavage of Notch receptor, nuclear translocation of NICD, and transcription activation in normal tissue (Callahan and Egan 2004). The proper timing of Notch pathway activation is also specified by events, such as proteolytic degradation, that control NICD levels in the nucleus. Mastermind recruits CycC:CDK8 to enhance phosphorylation and proteolytic turnover of NICD by facilitating PEST-dependent degradation through the Fbw7/Sel10 ubiquitin ligase (Fryer et al. 2004). Reelin stimulates Dab1 to protect NICD from Fbw7-induced degradation (Hashimoto-Torii et al. 2008). Itch is a cytoplasmic ubiquitin ligase that regulates the ubiquitination of Notch in a PEST-independent manner. The ankyrin repeat protein NRARP promotes NICD turnover in embryos in a PEST-independent manner (Lamar et al. 2001). Besides, AMPK stabilizes Notch1 by impairing the interaction between Notch1 and Itch in a kinase activity-dependent manner to potentiate hypoxia-induced breast cancer stemness and drug resistance (Mohini and Rangarajan 2018). Moreover, GSK3β acts positively within the Notch pathway in a kinase activity-dependent manner by protecting the NICD from proteasome-mediated degradation (Foltz et al. 2002). In this study, we provided evidence that NDR1 was a novel factor to modulate NICD protein stability. Interestingly, our data indicated NDR1 might mainly regulate the proteolytic turnover of NICD in an Fbw7-dependent manner. NDR1 competed with FBW7 for NICD interaction, thereby leading to stabilizing NICD.

NDR kinase family has been characterized for their roles in centrosome duplication (Hergovich et al. 2007), chromosome alignment (Chiba et al. 2009), cell polarity control (Yan et al. 2015) and cytoskeletal organization (Fang and Adler 2010). The kinase activity of NDR kinases is essential for exerting its biological functions (Tamaskovic et al. 2003). However, recent studies imply that NDR kinases might also function in a kinase activity-independent manner. Although NDR1 is critical for precise alignment of mitotic chromosomes (Chiba et al. 2009), whether this mitotic role depends on its kinase activity remains controversial (Cornils et al. 2011a). NDR1 might function as an adaptor molecule in mitosis independent of its kinase activity (Cornils et al. 2011a). Moreover, NDR1 stabilizes c-myc by interacting with c-myc and impairing c-myc ubiquitination (Cornils et al. 2011b). Nevertheless, both kinase-dead or wild type NDR1 interacts with and stabilizes c-myc at a similar level (Cornils et al. 2011b), indicating a kinase activity-independent role of NDR1 kinase. Consistently, our study indicated that NDR1 also interacted with and stabilized NICD in a kinase activity-independent manner.

In summary, our study revealed a previously unknown function and mechanism of NDR1 in regulating BCSC properties. Our study might provide a novel theoretical basis for designing strategies to target BCSC.

## Conclusions

Taken together, this study unveiled a novel mechanisms of aberrant activation of the Notch signaling pathway. To our knowledge, this study is the first to demonstrated that NDR1 promoted activation of Notch signaling by competing with Fbw7 for NICD binding to reduce the proteolytic turnover of NICD. Our study revealed a novel function of NDR1 in regulating BCSC properties by activating the Notch pathway. These data might provide a potential strategy for eradicating BCSC to overcome tumor relapses, metastasis and drug resistance.

## Supplementary Information

**Additional file 1: Fig. S1.** The effect of NDR1 on proliferation in breast cancer cells. **Fig. S2. **The effect of wild type or kinase dead NDR1 on CD24low/CD44high population in SUM149 cells. **Fig. S3.** ER stimulated BCSCs properties and Tamoxifen sensitivity might be not regulated by NDR1. **Fig. S4.** Activation of Notch1 signaling pathway is essential for NDR1 enhanced BCSC properties. **Fig. S5**. The effect of NDR1 on the expression of ADAM10/17 and Presenilin 1/2 in SUM149 cells. **Fig. S6.** Cut‐off plots.

## Data Availability

Not applicable.

## Abbreviations

BCSCs: Breast cancer stem cells
NICD: Notch intracellular domain
CBF-1: C-promoter binding factor 1
NDR: Nuclear-Dbf2-related
CHX: Cycloheximide
BSA: Bovine serum albumin
DEAB: Diethylaminobenzaldehyde
ALDH: Aldehyde dehydrogenase
OS: Overall survival
RFS: Relapse free survival
DMFS: Distant metastasis free survival
PPS: Post progression survival
