# Supplementary material for: NDR1 increases NOTCH1 signaling activity by impairing Fbw7 mediated NICD degradation to enhance breast cancer stem cell properties
Source: Mol Med. 2022 May 4;28:49. doi: 10.1186/s10020-022-00480-x (PMC9066784; doi:10.1186/s10020-022-00480-x)
Supplement: Supplementary file 1 — Additional file 1: Fig. S1. The effect of NDR1 on proliferation in breast cancer cells. Fig. S2. The effect of wild type or kinase dead NDR1 on CD24low/CD44high population in SUM149 cells. Fig. S3. ER stimulated BCSCs properties and Tamoxifen sensitivity might be not regulated by NDR1. Fig. S4. Activation of Notch1 signaling pathway is essential for NDR1 enhanced BCSC properties. Fig. S5. The effect of NDR1 on the expression of ADAM10/17 and Presenilin 1/2 in SUM149 cells. Fig. S6. Cut‐off plots. [file 10020_2022_480_MOESM1_ESM.zip › Esm/Supplementary Figure Legends.docx]

**Additional file 1 Figure Legends**

Fig. S1. The effect of NDR1 on proliferation in breast cancer cells.

**a** Exponential growth breast cells were harvested, and subjected to western blot analysis. **b** NDR1 or control vector were transfected in SUM149 or MCF-7 cells. Cells were harvested at day 3 or 5, and subjected to western blot analysis. **c, d** NDR1 or control vector were expressed in SUM149 or MCF-7 cells. Then, cells were plated and subjected to cell counting at indicated time. **e** NDR1 siRNA or control siRNA were transfected in SUM149 or MCF-7 cells. Cells were harvested at day 3 or 5, and subjected to western blot analysis. **f, g** NDR1 siRNA or control siRNA were transfected in SUM149 or MCF-7 cells. Then, cells were plated and subjected to cell counting at indicated time. **h** The representative ﬂow cytometry results of Fig. 1a. The experiment setting was the same as Fig. 1a. **i** The representative ﬂow cytometry results of Fig. 1b. The experiment setting was the same as Fig. 1b. **j** The representative ﬂow cytometry results of Fig. 1e. The experiment setting was the same as Fig. 1e. **k** The representative ﬂow cytometry results of Fig. 1f. The experiment setting was the same as Fig. 1f. **l** SUM149 was treated with indicated concentration of Epirubicin for 48 hours. Then cells were harvested for Annexin V-FITC staining and ﬂow cytometry analysis. **m** MCF-7 was treated with indicated concentration of Epirubicin for 48 hours. Then cells were harvested for Annexin V-FITC staining and ﬂow cytometry analysis. **n** The representative ﬂow cytometry results of Fig. 1g. The experiment setting was the same as Fig. 1g. **o** The representative ﬂow cytometry results of Fig. 1h. The experiment setting was the same as Fig. 1h. The bar represents mean ± SD of three independent experiments (#: p>=0.05).

Fig. S2. The effect of NDR1 on CD24low/CD44high population in breast cancer cells.

**a-h** The representative ﬂow cytometry results of Fig. 2a-2h. The experiment setting was the same as Fig. 2a-2h. **i, j** Wild type (WT) or kinase-dead (KD, K118A) NDR1 was overexpressed for 72 hours in SUM149 cells. Cells were harvested and subjected to western blot analysis or ﬂow cytometry analysis of CD24/44 expression. The bar represents mean ± SD of three independent experiments (*: p<0.05, **: p<0.01, ***: p<0.001).

Fig. S3 ER stimulated BCSCs properties and Tamoxifen sensitivity might be not regulated by NDR1.

**a, b** MCF-7 or SUM149 cells were treated with estradiol (1 nM) or ethanol for 72h. Cells were harvested for ﬂow cytometry analysis of CD24/44 expression (a) and western blot analysis (b). **c, d** NDR1 or control vector were expressed in MCF-7 or SUM149 cells. Then, cells were treated with or without Tamoxifen (OHT, 5μM). Cell number were counted at indicated time. The bar represents mean ± SD of three independent experiments (#: p>=0.05, *: p<0.05, **: p<0.01, ***: p<0.001).

Fig. S4 Activation of Notch1 signaling pathway is essential for NDR1 enhanced BCSC properties.

**a, b** NDR1 or control vector was expressed in MCF-7 cells. Cells were treated with the indicated concentration of DAPT for the indicated time. Cells were harvested and subjected to CD24/44 analysis (72 hours, a) and sphere-forming assay (7 days, b). The distribution of sphere diameters was shown in b. The bar represents mean ± SD of three independent experiments (#: p>=0.05, *: p<0.05, **: p<0.01, ***: p<0.001).

Fig. S5. The effect of NDR1 on the expression of ADAM10/17 and Presenilin 1/2 in SUM149 cells.

**a** NDR1 or control vector were expressed in SUM149 cells for 48 hours. Then, cells were subjected to western blot analysis. **b** NDR1 siRNA or control siRNA were transfected in SUM149 cells for 48 hours. Then, cells were subjected to western blot analysis.

Fig. S6 Cut-off plots

**a-d** Cut-off plots used to visualize the correlation between the used cut-off values and the achieved P values and hazard rate (HR). The red circle identified the best cutoff.
