# Supplementary figures and images for: NDR1 increases NOTCH1 signaling activity by impairing Fbw7 mediated NICD degradation to enhance breast cancer stem cell properties
Source: Mol Med. 2022 May 4;28:49. doi: 10.1186/s10020-022-00480-x (PMC9066784; doi:10.1186/s10020-022-00480-x)

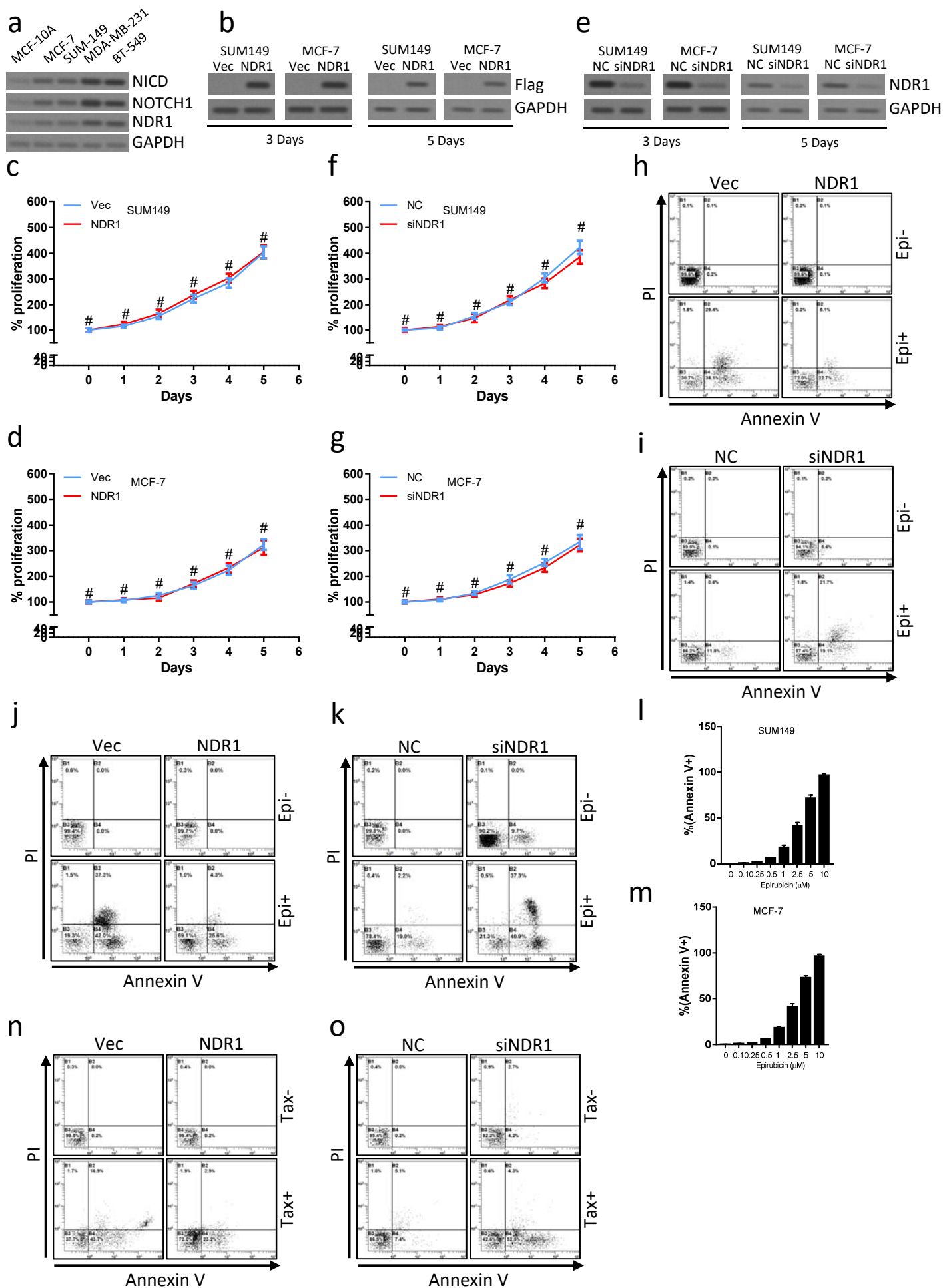

Fig. S1. The effect of NDR1 on proliferation in breast cancer cells.

Supplement: Supplementary file 1 — Additional file 1: Fig. S1. The effect of NDR1 on proliferation in breast cancer cells. Fig. S2. The effect of wild type or kinase dead NDR1 on CD24low/CD44high population in SUM149 cells. Fig. S3. ER stimulated BCSCs properties and Tamoxifen sensitivity might be not regulated by NDR1. Fig. S4. Activation of Notch1 signaling pathway is essential for NDR1 enhanced BCSC properties. Fig. S5. The effect of NDR1 on the expression of ADAM10/17 and Presenilin 1/2 in SUM149 cells. Fig. S6. Cut‐off plots. [file 10020_2022_480_MOESM1_ESM.zip › Esm/Supplementary Figure 1.pdf]

a

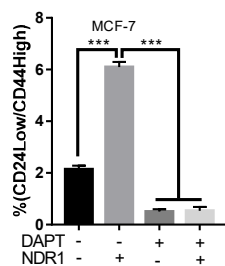

b

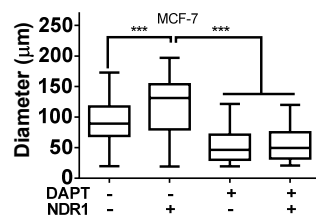

Fig. S4. Activation of Notch1 signaling pathway is essential for NDR1 enhanced BCSC properties.

Supplement: Supplementary file 1 — Additional file 1: Fig. S1. The effect of NDR1 on proliferation in breast cancer cells. Fig. S2. The effect of wild type or kinase dead NDR1 on CD24low/CD44high population in SUM149 cells. Fig. S3. ER stimulated BCSCs properties and Tamoxifen sensitivity might be not regulated by NDR1. Fig. S4. Activation of Notch1 signaling pathway is essential for NDR1 enhanced BCSC properties. Fig. S5. The effect of NDR1 on the expression of ADAM10/17 and Presenilin 1/2 in SUM149 cells. Fig. S6. Cut‐off plots. [file 10020_2022_480_MOESM1_ESM.zip › Esm/Supplementary Figure 4.pdf]

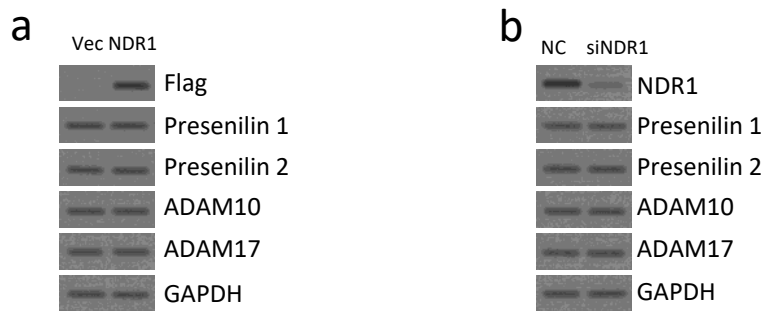

Fig. S5. The effect of NDR1 on the expression of ADAM10/17 and Presenilin 1/2 in SUM149 cells.

Supplement: Supplementary file 1 — Additional file 1: Fig. S1. The effect of NDR1 on proliferation in breast cancer cells. Fig. S2. The effect of wild type or kinase dead NDR1 on CD24low/CD44high population in SUM149 cells. Fig. S3. ER stimulated BCSCs properties and Tamoxifen sensitivity might be not regulated by NDR1. Fig. S4. Activation of Notch1 signaling pathway is essential for NDR1 enhanced BCSC properties. Fig. S5. The effect of NDR1 on the expression of ADAM10/17 and Presenilin 1/2 in SUM149 cells. Fig. S6. Cut‐off plots. [file 10020_2022_480_MOESM1_ESM.zip › Esm/Supplementary Figure 5.pdf]

a

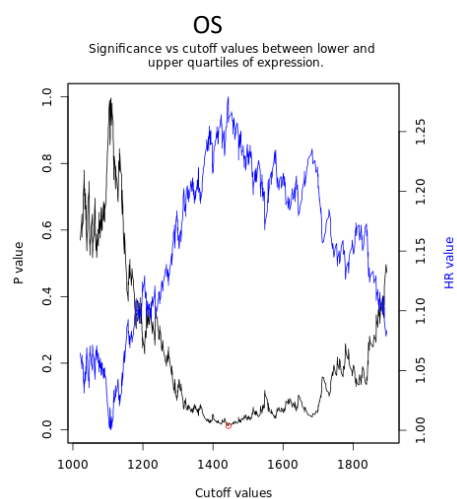

b

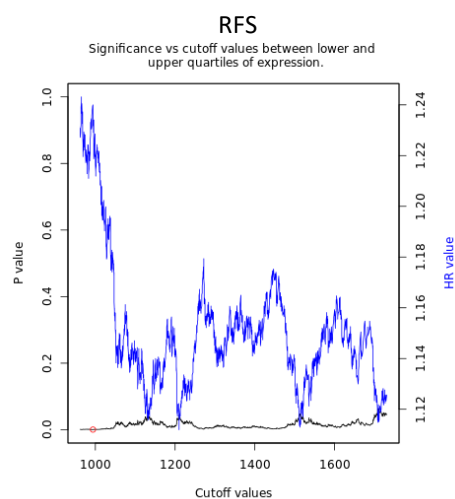

c

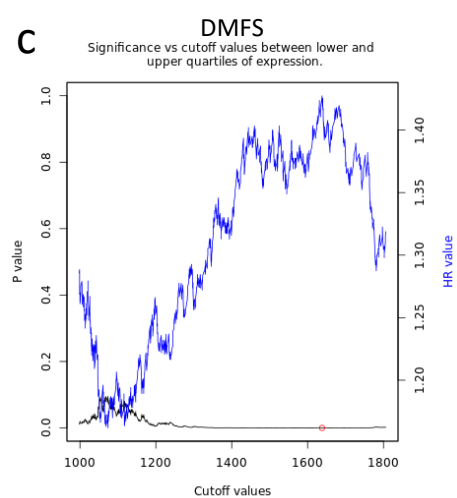

d

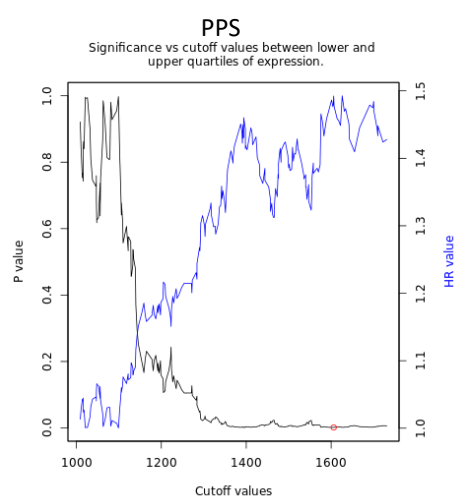

Fig. S6. Cut-off plots.

Supplement: Supplementary file 1 — Additional file 1: Fig. S1. The effect of NDR1 on proliferation in breast cancer cells. Fig. S2. The effect of wild type or kinase dead NDR1 on CD24low/CD44high population in SUM149 cells. Fig. S3. ER stimulated BCSCs properties and Tamoxifen sensitivity might be not regulated by NDR1. Fig. S4. Activation of Notch1 signaling pathway is essential for NDR1 enhanced BCSC properties. Fig. S5. The effect of NDR1 on the expression of ADAM10/17 and Presenilin 1/2 in SUM149 cells. Fig. S6. Cut‐off plots. [file 10020_2022_480_MOESM1_ESM.zip › Esm/Supplementary Figure 6.pdf]
